# Was the Cinta Senese Pig Already a Luxury Food in the Late Middle Ages? Ancient DNA and Archaeozoological Evidence from Central Italy

**DOI:** 10.3390/genes11010085

**Published:** 2020-01-11

**Authors:** Federica Gabbianelli, Francesca Alhaique, Giuseppe Romagnoli, Luca Brancazi, Lavinia Piermartini, Claudio Ottoni, Alessio Valentini, Giovanni Chillemi

**Affiliations:** 1 DIBAF, University of Tuscia, 01100 Viterbo, Italy; alessio@unitus.it; 2 Bioarchaeology Service, Museum of Civilizations, 00118 Rome, Italy; 3 DISTU, University of Tuscia, 01100 Viterbo, Italy; romagnoli@unitus.it (G.R.); laviniapiermartini@gmail.com (L.P.); 4 PhD School of Archaeology, Post-Classical Archaeology, Sapienza University, 00118 Rome, Italy; luca.brancazi@uniroma1.it; 5 Department of Oral and Maxillofacial Sciences, Diet and Ancient Technology Laboratory (DANTE), Sapienza University, 00118 Rome, Italy; claudio.ottoni@uniroma1.it; 6 Institute of Biomembranes, Bioenergetics and Molecular Biotechnologies, IBIOM, CNR, 70121 Bari, Italy

**Keywords:** *Sus scrofa*, *KIT*, sexing markers, wild boar, introgression

## Abstract

The Cinta senese is a pig breed, highly esteemed for its meat and derived products, characterized by a black coat with a typical white “belt” and documented by scant iconography, since the 13^th^–14^th^ century in Italy. A piece of pottery showing a Cinta pig was found in the Graffignano castle (Northern Latium, Italy) dated 15th–16th centuries, spurring us to investigate the diet of the inhabitants. Ancient DNA analysis was carried out on 21 pig specimens on three nuclear SNPs: (1) g.43597545C>T, on the *KIT* gene, informative for the identification of the Cinta senese breed; (2) rs81460129, on an intergenic region in chr. 16, which discriminates between domestic pigs and wild boars, and; (3) a SNP on the *ZFY*/*ZFX* homologous genes, to determine the sex of the individuals. Our results indicate that the Cinta senese was present in Northern Latium in Late Medieval time, although it was not the only breed, and that pigs, including Cinta, interbred with wild boars, suggesting free-range breeding for all types of pigs. Moreover, the unexpected high proportion of young females may be considered as evidence for the wealth of the family inhabiting the castle.

## 1. Introduction

Plant and animal domestication represented a dramatic change in human evolution, determining the shift to food-producing subsistence strategies. Over the past few years, several multidisciplinary studies have tackled the modes and times of dispersal of animal domesticates along with humans, as well as the selective processes that grounded the profound physical and behavioral changes that differentiate wild and domestic animals [1]. Ancient DNA (aDNA), in particular, has proven to be an invaluable tool to analyze genetic variation across time, and investigate past evolutionary processes otherwise undetectable through the analysis of modern populations alone, such as bottlenecks, admixture with wild populations, and dispersals [2].

Pigs (*Sus scrofa*) were domesticated for the first time around 11,000–10,000 years ago (ya) in Southwest Asia, together with sheep, goats and cattle [3,4,5,6,7,8,9]. A second independent domestication of pigs occurred about 8000 ya in East Asia [10,11]. Zooarchaeological and aDNA studies have shown that pig domestication was a complex process, involving multiple dispersals across West-Eurasia [12] and long-term admixture between wild and domestic populations [13]. Starting most likely from the late medieval-early modern era, a shift from extensive farming to more intensive rearing of pigs in confinement occurred, as documented by morphological changes in domestic stocks [14,15]. More intentional breeding programs started from the 17^th^ century [16,17], and continued more actively over the last century with the creation of common commercial breeds.

Nowadays, the economic value of pigs is doubtless, and pork is one of the most consumed types of meat worldwide. European pig breeds are distinguished between global commercial breeds, and local heritage breeds that were not hybridized with Asian pigs during the Industrial Revolutions. Growing attention is being focused on understanding the evolutionary processes behind breed formation and their use in agricultural exploitation. Technological advancement and recent findings in genomics to investigate pedigrees have drastically changed animal breeding, with particular regard to selection of specific desirable traits, genetic progress maintenance, and large-scale industrial meat production [18,19]. Moreover, the knowledge of which breeds were present in a particular location and time will help to gather information on the type of husbandry and of commodities obtained/traded [20]. So far, most studies of animal breed origin focused on modern populations [21,22,23], inferring past breed features through markers of extant breeds [24,25] or iconography and morphological markers [26,27]. Only a few papers have investigated livestock breeds using ancient genetic data [28,29,30,31].

**Figure 1:**
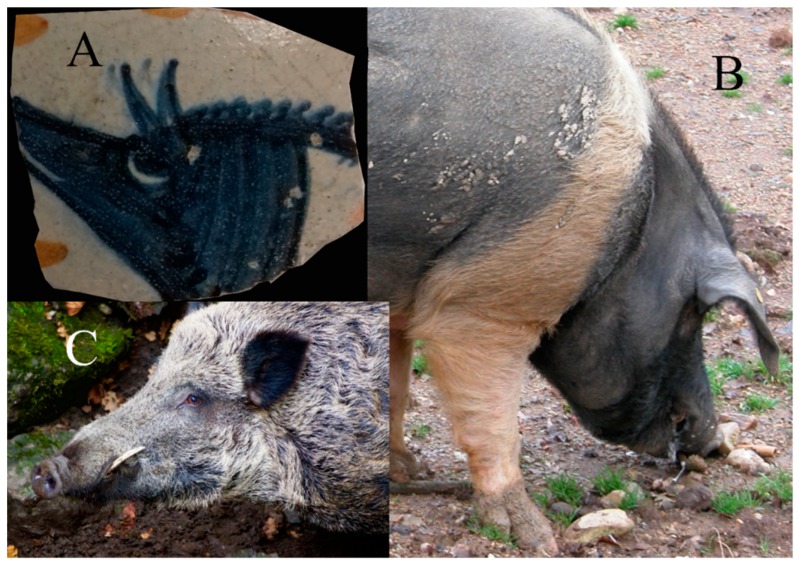
Representation of pig vs. extant Cinta senese breed and wild boar. (**A**) Medieval ceramic fragment retrieved from pit 2 at the Graffignano Castle. The represented pig has a white belt and long snout. (**B**) Cinta senese picture. The typical white belt is evident. (**C**) Wild boar picture. The snout is longer than in *S. scrofa*.

Here, we report the aDNA analysis on Late Medieval pig bone samples collected from the castle of Graffignano, in Central Italy. The town of Graffignano is located in the Tiber Valley about 60 km south of the border with Tuscany, in the northern part of Latium, and was built during the 12^th^ or the 13^th^ century CE. The castle was acquired and renewed by the Baglioni family in the last quarter of the 15^th^ century, by applying the criteria of early Renaissance fortified architecture. We conducted archaeological excavations on the ground floor between 2009 and 2011 in 2 refuse pits (locally called “butti” or “pozzi da butto”) carved in the volcanic tuff bedrock. Among all the tableware in polychrome maiolica, lusterware and graffita pottery used by the Baglioni family recovered from the pits, we found a fragment of a polished ceramic jug, dated to the beginning of the 16^th^ century, with the representation of a “belted” pig figure (Figure 1a), similar to the more famous one appearing in the Allegoria del Buon Governo by Ambrogio Lorenzetti of the Palazzo Comunale in Siena (1338).

Cinta senese is a belted pig breed currently highly esteemed for its meat and derived products. Since 2012 it obtained the Protected Designation of Origin (PDO) status, one of the European Union schemes to protect geographical indications and traditional specialties. This breed is documented by scant iconography starting from the 13^th^–14^th^ century in Italy, such as the Allegoria del Buon Governo by Ambrogio Lorenzetti (Palazzo Comunale di Siena, 1338 CE) and the statuary group representing December in the archivolt of the main entrance of Santa Maria della Pieve (Arezzo, 1216 CE). Most of the instances are located in Tuscany and, to the best of our knowledge, no documentation has been found so far in Latium dated in the same period or afterwards. The coat color of Cinta Senese is black with a typical white belt. The belt can be found in different shapes also in other modern breeds (e.g., Hampshire and Poland China). Morphological analyses of archaeological remains do not offer sufficient resolution to assess coat color phenotypic information, hence, it is of crucial importance for our study to investigate coat color genes. Here we analyzed a SNP on the *KIT* gene, which is diagnostic for Cinta senese breed. The *KIT* gene encodes the mast/stem cell factor receptor (SCFR) and transduces an intracellular signal through tyrosine kinase activity [32]. The cellular gene *c-KIT* is homologous to the transforming gene *v-KIT* from a feline retrovirus [33]. The SCFR activity is essential to melanoblast proliferation, survival and differentiation into melanocytes [34].

The aims of the study are: (i) to investigate the presence of the Cinta senese (or a similar one) pig breed in Late Medieval times in Northern Latium; (ii) to determine if in this region pigs interbred with wild boars, and (iii) to assess ratio between male and female individuals.

## 2. Materials and Methods

### 2.1. Archeological Excavation

Refuse pit 1 presents a biconical section with a flat bottom and is about 2.95 m deep, while refuse pit 2 is flask shaped with a flat bottom, 3.55 m deep. They were most likely used as silos or cisterns and then reused as pits to dump garbage produced by the residence, like ceramic, glass and metal objects (consumed, broken or considered outmoded), but also animal remains (mainly meal leftovers) and other items accidentally fallen.

The ceramic artifacts found inside pit 1 (211 shards) belong to the northern Latium production referable to the first quarter of the 15^th^ century: tin-glazed pottery (*maiolica arcaica*), lead-glazed pottery (*graffita*) and common wares.

Pit 2 was filled in two phases: in the first period (circa 1425–1450) broken or consumed materials, also of fine quality (*maiolica arcaica* and *zaffera* mugs, bowls and basins) were gradually cleared out. The second phase of the filling (before circa 1500–1525) is constituted by ceramics and building materials, most likely in relation to the renovation of the castle. As far as the pottery is concerned, Pit 2 contained a total of 1451 ceramic fragments dated on the basis of typology and decoration between the end of the 14th and the beginning of the 15^th^ century.

An analysis of the ceramic material collected allowed the almost complete reconstruction of the table and serving sets used in the castle (e.g., jugs, mugs, cups, bowls, small dishes, and large serving plates) in addition to pots used for cooking, preparing or preserving food and other domestic uses. Besides the pottery from renowned production centers in the nearby cities of Viterbo and Orvieto, there are also high quality ceramic artifacts, probably commissioned *ad hoc*, from workshops in Deruta, a town where the Baglioni family owned a palace and probably also a pottery kiln, documented to ca. 1450 [35,36].

### 2.2. Archaeozoological Materials and Methods

The faunal assemblage from the Graffignano castle includes 54 specimens from pit 1 and 1502 from pit 2. The assemblage is relatively fragmented, but the preservation of bone surfaces is usually quite good; all specimens, including the unidentifiable ones, were inspected in order to identify human, animal, and other natural modifications. The age of the domestic species identified was calculated on the basis of archaezoological literature [37,38,39,40,41,42]. Withers height of the pig was estimated using [43].

### 2.3. Sample Selection

Archaeological samples used for aDNA analysis were taken during the excavation to minimize sample contamination and degradation before storage, as previously described [44].

The sampled pig specimens belong to different individuals identified on the basis of age, size, sex and skeletal element [35]. The samples were collected from different layers along the stratigraphic sequences of the two pits, two out of a minimum number three individuals from refuse pit 1 and 19 out of 30 animals from refuse pit 2 (Table 1). The dating of the skeletal elements was made on the basis of the typology and decoration of the ceramic materials, retrieved in the same layers.

### 2.4. DNA Extraction

For the selected 21 pig skeletal elements, aDNA was extracted from bones in the dedicated aDNA laboratory facilities of the University of Tuscia, following strict precautions commonly described in the literature (e.g., [45]) such as: isolation of work areas; drilling of one single sample a day; use of negative control extraction and amplification, and; at least 2 independent extractions and amplification.

Bone samples were pulverized with a drill, after removing the outer surface with sandpaper. In order to reduce contamination from the external surface and to preserve the bone for future morphological studies, we perforated the bone and dug internally. To prevent cross-contamination, only a sample per day was processed. For each sample, bone powder (about 500 mg) was incubated in 2 mL extraction buffer (0.5 M EDTA pH 8.0, 0.5% sodium dodecyl sulfate, and 100 µg/mL proteinase K) at 55 °C overnight and then at 37 °C for 24 h. DNA was purified with silica QIAquick column (Qiagen, Düsseldorf, Germany) (Yang’s protocol, [46]). Every extraction batch was composed of five samples and one extraction blank.

DNA extracted from ancient samples as well blank controls was quantified using a Qubit Fluorometer (ThermoFisher Scientific, Waltham, MA, USA) according to the manufacturer’s instructions (Table 2). To increase the extraction yields of samples with no DNA or very low concentration (i.e., G8, G21, G24, G182) we also used a different extraction method (Dabney’s protocol, [47]), with slight modifications [48].

### 2.5. Amplification and Sequencing

A fragment of 157 bp in the *KIT* gene, determining the coat color, was amplified through standard PCR to detect the informative SNP g.43597545C>T (chromosome 8 of the Sscrofa10.2 genome assembly). This polymorphism is different from other known *KIT* mutations in pigs (see Table 1 in [34]), and it is nearly fixed in Cinta senese (T allele frequency equal to 95.9%), whereas allele C is present in non-belted breeds [49,50]. Primer sequences, developed in this study, are: 5′-TGAACATTGCTGACTCCCCT-3′ (forward); 5′-TGCATTTTACCTAAAGAGAAGAGC-3′ (reverse). A 5 min denaturation step at 95 °C was followed by 35 cycles of denaturation at 95 °C (30 s), annealing at 56 °C and extension at 72 °C (30 s), the final extension step was carried out at 72 °C for 10 min.

In order to assess the wild (WBO: Wild Boar) or domestic status (DP: Domestic Pig) of the animals investigated, we amplified a 156 bp-long fragment on chromosome 16 containing a diagnostic SNP (rs81460129), reported at an allelic frequency of 0.95 both for domestic (T) and WBO (C) [51]. In this respect, we are making the assumption that the allele frequency in WBO and DP has not changed significantly from medieval times. In fact, taking into account that: (i) mutations are negligible in this time span, (ii) animals had not more than 250 generations, (iii) no major bottlenecks were expected since the sub-species are highly prolific and mobile in a wide areal range with no barriers, and (iv) artificial selection started only very recently and mostly on cosmopolitan breeds rather than on WBO and Cinta Senese, we feel rather confident that the assumption might hold. Primer sequences, developed in this study, are: 5′-GAAAGGCAGGACKTGAGTGTC-3′ (forward); 5′-TCRAGCTCCTGCTCACTAAT-3′ (reverse). A 5-min denaturation step at 95 °C was followed by 20 cycles of denaturation at 94 °C (30 s), annealing starting from 61 °C and decreasing 0.5 °C per cycle (30 s) and extension at 72 °C (1 min), then by 20 cycles of denaturation at 94 °C (30 s), annealing at 54 °C (30 s) and extension at 72 °C (1 min); the final extension step was carried out at 72 °C for 10 min.

We designed suitable primers for aDNA to amplify a SNP on a short fragment of *ZFY*/*ZFX* genes and discriminate male samples from female ones. Primers included a portion of both *ZFY* (X75511) and *ZFX* (X75510) genes of 209 bp. Primer sequences are: 5’-AAGGAGCCAACAAAATGCAC-3’ (forward); 5’-TTCGTCACCCATCAGAGCTC-3’ (reverse). A 5-min denaturation step was followed by 20 cycles of denaturation at 94 °C (30 s), annealing starting from 61 °C and decreasing 0.5 °C per cycle (30 s) and extension at 72 °C (1 min), then by 20 cycles of denaturation at 94 °C (30 s), annealing at 54 °C (30 s) and extension at 72 °C (1 min); the final extension step was carried out at 72 °C for 10 min.

In all the analyses we amplified the DNA using 0.5 pM of each primer (Sigma-Aldrich, St. Louis, MO, USA), 10× Optibuffer, 50 mM MgCl2 solution, dNTPs 10 mM, 4U/µL of BIO-X-ACT short (Bioline, London, UK) in a final volume of 20 µL.

All PCR products were analyzed in a laboratory dedicated to the analysis of modern DNA samples, physically separated from the aDNA facilities. PCR products were visualized on a 2.2% FlashGel (Lonza, Basilea, Switzerland) and purified with ExoSAP-IT (ThermoFisher Scientific, Waltham, MA, USA), according to the manufacturer’s instructions and sent to Eurofins Genomic for sequencing.

### 2.6. Genotyping with PCR-RFLP

We used also a second independent method to discriminate between Cinta senese pigs and other pigs without the white belt. We designed primers to amplify by PCR a shorter portion of 99 bp of the *KIT* gene including the SNP g.43597545C>T. Primer sequences are: 5′-GACTCCCCTGTGCTTCCACT-3′ (forward); 5′-CCAGACATCGCTTTCAAATGTGT-3′ (reverse).

We amplified the DNA using 10 ng DNA, 0.5 pM of each primer (Sigma-Aldrich, St. Louis, MO, USA), 10× Optibuffer, 50mM MgCl2 solution, dNTPs 10 mM, 1 U of BIO-X-ACT short (Bioline, London, UK) in a final volume of 20 µL. A 5-min denaturation step at 95 °C was followed by 35 cycles of denaturation at 95 °C (30 s), annealing at 56 °C and extension at 72 °C (30 s), the final extension step was carried out at 72 °C for 10 min.

An aliquot of the PCR mix was used to amplify samples of modern breeds as positive controls for each aDNA run, in another laboratory dedicated to modern DNA. PCR products were visualized and evaluated on a 2.2% FlashGel (Lonza, Basilea, Switzerland).

Amplification products were digested with the restriction enzyme DdeI (NEB). For each digestion, 5 µL of amplification product was added to a mix of enzyme (10 u/µL), buffer (1X) and H2O to a final volume of 50 µL, then incubated at 37 °C overnight in a thermocycler. Reactions were stopped by heating at 65 °C for 20 min, according to enzyme manufacturer instructions. The digestion produced two fragments when the amplicon contained allele T and only one when allele C was present. The different alleles were scored on a 2.2% FlashGel (Lonza, Basilea, Switzerland).

## 3. Results

### 3.1. Archaeozoological Analysis

The archaeozoological analysis of the faunal assemblages recovered in the two pits of the Graffignano Castle helped us to reconstruct the meat component of the diet of the people living in the castle as well as some information on the discard practices.

**Figure 2:**
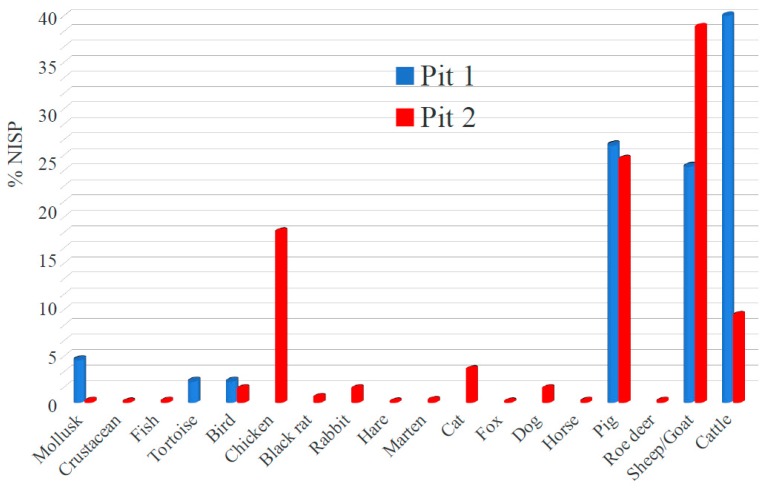
Species composition and proportion of the faunal samples from the two pits. The NISP (Number of Identified Specimens) is 1319 and 45 in pit 2 and pit 1, respectively, not including the unidentifiable fragments.

The species composition and proportion in the faunal samples from the two pits are quite different (Figure 2). In pit 1 cattle is dominant, followed by pig and sheep/goat, the latter two with similar proportions; while in pit 2 sheep/goat is the prevalent taxon, pig is the second species, and cattle the third. However, in both cases cattle provided the highest meat yield, followed by pigs and then by sheep/goat. The large assemblage from pit 2 indicates that the diet was implemented by a wide array of species including chicken and other birds, lagomorphs, aquatic resources (molluscs, crustaceans, fish), and even a pine marten, as documented by cut marks identified on the femur and the tibia; while in pit 1 a tortoise, an eggshell, and a freshwater mollusc were also found.

The age determination of the main domestic species shows that in pit 2 there was a prevalence of very young individuals for sheep/goat, pigs, as well as chicken, while in pit 1 the few animals recovered were older (Table 2 and [36]).

Bone modifications (e.g., cut marks, chop marks, burning) detected on the specimens suggest that most of the bones in both pits represents food debris. Moreover, pit 2 was used also to discard the bodies of pets (dog and cat) and pests (rats), as well as parts of other animals likely not used as food (e.g., horses, foxes, a roe deer antler) because they do not show bone modifications related to butchery for consumption.

The adult pigs of pit 2 show a relatively large size (withers height ca. 80–82 cm), and the shape of the lacrimal bone, a diagnostic morphological feature to discriminate wild and domestic swine [52], is relatively elongated suggesting possible recent interbreeding with WBO, most likely related to free range herding [53,54].

### 3.2. Genetic Analysis

We selected 21 bones, belonging to different individuals, of which 17 produced quantifiable DNA with an average of 4 ng per g of powder bone, as from Qubit data (see Materials and Methods).

For the four remaining samples, higher DNA extraction yields were obtained with the second method, which was demonstrated to significantly increase the efficiency of aDNA recovery [47,48]. For this reason, we used this method for independent DNA extraction replicates on samples identified as “cinta”, and their recovery rate increased with an average of 6 ng per g of powder bone.

#### 3.2.1. Assessing the Presence of Cinta Senese Breed

All samples were successfully checked for the *KIT* SNP (note that the T allele typical of the Cinta senese [49,50]) with two independent PCR protocols that produced the same results (see 2.5, 2.6 and Table 2). Seventeen samples resulted as homozygous for the C allele, two homozygous for the T allele and two heterozygous (C/T). Since the *KIT* mutation is dominant, the pigs heterozygous for this mutation are also belted. Therefore, we were able to confirm the presence of the Cinta senese breed allele in Northern Latium in the Late Medieval time, with a frequency in our sample of 16%.

#### 3.2.2. Detection of Possible Cross-Breeding

**Figure 3:**
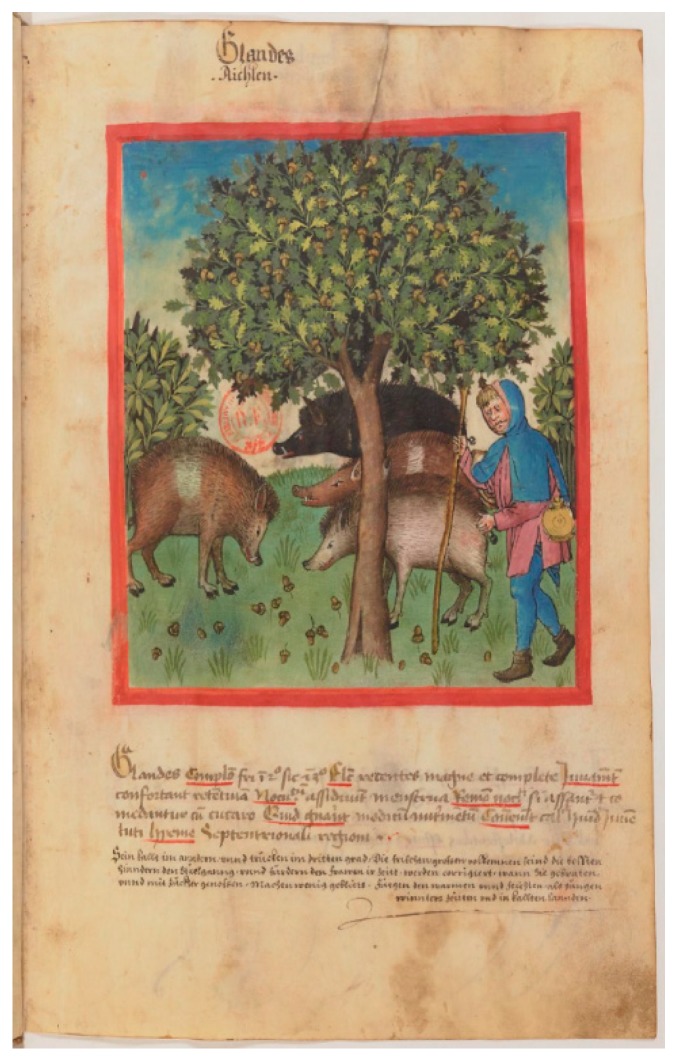
Pig herding scene from the Tacuinum Sanitatis (BnF Ms.Lat.933). Different breeds are present in the same herd. (Source gallica.bnf.fr / Bibliothèque nationale de France)

Since livestock keeping in Medieval times in Italy by and large consisted of free range pasture, we aimed to analyze whether domestic pigs (DP) have been crossed with WBO, purposely to increase robustness or by chance. This is also suggested: (i) by our ceramic fragment (Figure 1A), which depicts a belted pig (Figure 1B) with an elongated snout typical of WBO (Figure 1C); (ii) by other pig images of this period (see Figure 3); (iii) by the large size of the adult pigs from Graffignano pits, and; (iv) by the shape of the lacrimal bone of some specimens (see 3.1). Therefore, we analyzed a SNP which discriminates between WBO and DP [55] (see 2.5). This SNP has a C allele with a frequency of 0.95 in WBO and of 0.05 in DP [51]. In this case, 15 of the 21 samples were successfully amplified and sequenced (Table 2). One of these samples (i.e., G133) showed the C allele, typical of WBO, and it was heterozygous at *KIT* gene. This result indicates a possible cross of Cinta senese with WBO, as suggested also by the iconography (Figure 1A). Three samples identified as “not Cinta”, G264, G23 and G24, showed the C allele, indicating that the crossing was not limited to the Cinta breed.

#### 3.2.3. Sexing of the Bones

We choose the *ZFX*/*ZFY* genes that are located in the homologous X and Y chromosome region [44]: 11 of the 21 samples were successfully amplified and sequenced (Table 2), showing lower amplification yields than the shorter fragments analyzed in the *KIT* genes, as expected for authentic ancient DNA following diagenetic trajectories (e.g., [45]). Only two of these have been diagnosed as males, indicating an interesting and unexpected prevalence of females, not only among the adult individuals, but also in the juvenile and very young age cohorts.

## 4. Discussion

We demonstrate the presence in Late Medieval times in Northern Latium of a pig breed similar, if not equal, to the extant one still actively exploited in Central Italy, namely the Cinta senese (“Belted from Siena”). Moreover, we show that in this region pigs interbred with wild boars, most likely as a result of common Medieval free-range herding practices. This is in agreement with recent genomic and archaeozoological evidence suggesting that long-term gene flow between wild and domestic animal populations was common [53,56]. The archaeozoological analysis showed that most of the animals were butchered at young age. Furthermore, most specimens sequenced at *ZFY*/*ZFX* homologous genes were diagnosed as females. The above results and the richness of the archaeological findings of the pits indicates the affluence of the inhabitants of the castle.

Morphological analyses of archaeological remain do not allow to assess coat color phenotypic information, so it is important to investigate coat color genes. Due to possible allelic drop-out, especially in ancient degraded samples, we are wary of defining homozygous genotypes. Nevertheless, we verified the presence of the Cinta allele, located in the small region responsible for the belted phenotype typical of this breed, in at least four individuals. Therefore, we are confident we have demonstrated the presence of Cinta senese in northern Latium, close to Tuscany, about 500 years ago both with aDNA data and with iconographic evidence. In particular, the fragment of ceramic jug found in the same pit 2 of the Graffignano castle in which the Cinta bone samples were retrieved (see Table 2).

Interestingly, the same breed is conserved today in the same territory and it is particularly appreciated and sold at prime prices. Cinta is one of the very few breeds that, thanks to its high quality features, has not been pushed towards extinction by the cosmopolite breeds used by the industry.

These results are interesting because often the current breeds are not descended from the old landrace breeds present in the area, but were imported or cross-bred [57,58]. However, in our study there is evidence that the foundations of the current Cinta pigs actually trace back at least to the Medieval ages. It is important to preserve these rare breeds because they are part of our heritage and for the maintenance of genetic diversity of the species. Furthermore, in the past the majority of aDNA studies have concentrated on the mtDNA, but because of the widespread introgression from the wild boar, mostly males, this is not a very good marker to study the history of the pig species. Instead, our study is based on nuclear markers that are sex-independent and in much higher number allowing to choose the more suitable ones.

In addition, we wondered about the elongated snout which is more similar to that of wild boar (Figure 1C) [59]. Moreover, the shape of two lacrimal bones retrieved in the same pit is relatively elongated as in the wild sub-species and a few measurable specimens indicate the presence of large sized animals. Therefore, we further checked for a SNP marker that discriminates between the wild and domestic taxon [51]. The results showed that in Northern Latium, in Medieval times, pigs still interbred with WBO, as already found in other European locations [56,60]. The iconography of Medieval pigs also indicates a more WBO-like appearance compared to modern breeds. Furthermore, ancient depictions (Figure 3) suggest the presence of different breeds within the same herd, as also corroborated by the genetic analysis in this study.

Interestingly, one individual has alleles in both loci (*KIT* and WBO) typical of Cinta and WBO. Since the belted allele is dominant [61] the individual would have appeared as belted, but probably the WBO contribution could have determined the longer snout.

Furthermore, we analyzed *ZFY*/*ZFX* homologous genes to determine the sex of the samples and nine individuals out of eleven successfully sequenced turned out to be females. Notably females are more tender than males and since they do not carry the “boar taint” smell [62] they have better meat quality than males unless castrated [63], which might have been cumbersome in Medieval times due to scarce hygienic conditions. The age determination for the pigs evidenced the prevalence of young and very young animals over adult ones with a ratio of 3:2. Again, it is well known that young animals are more tender and more palatable than old ones.

From zooarchaeological point of view, pigs are usually sexed on the basis of the shape and size of canines, but these features cannot be easily distinguished in young and very young individuals. As a consequence detailed comparative data for similar assemblages (i.e., with high proportions of young animals) are lacking in the literature, assessment of sex ratio in relation to age at butchering is very rarely reported and often only in general terms, although a selection of younger male pigs is suggested in Italy since Roman times (e.g., [64]).

In this study, by using aDNA techniques, we have obtained a clear picture of the sex ratio. Surprisingly, it is quite the opposite of what was suggested by Medieval sources (e.g., [65]), strongly influenced by classical authors such as Columella, Varro, and Palladio, who contended the optimal ratio of 10 males to 100 females in a pig herd. This ratio could be obtained by killing surplus young males; sows were instead kept alive for reproduction until a more mature age and would be killed earlier only if not productive. Therefore, the resulting expectation in a faunal assemblage from food debris would be a prevalence of males in the younger age classes. However, we have found remains of young, and even very young females, meaning that they have been butchered even before knowing their productivity. A possible explanation for “spoiling” this resource of future economic return could be the (further) ostentation of wealth that was already clear from the context and other archaeological findings.

## 5. Conclusions

The use of aDNA techniques and established archaeological tools allowed us to shed light on the life scenario of a wealthy family of Medieval Ages.

We discovered that female individuals, often slaughtered at a young age were part of their diet. Usually females were exploited for reproduction, but the Baglioni family ignored the costs of killing a reproductive sow at young age in favor of splurging in luxury. We might infer that the taste of the family was attracted by tender, juicy, yet tasty meat, as it is also reflected by the young age of sheep/goat and even chicken. On the other hand, genetic data seem to suggest that, in contrast to the present-day situation in which local animal genetic resources are protected, the Cinta breed was only one of those exploited during this period, and its “pureness” was not considered a priority within the managing strategies, as evidenced by the presence of cross-breeding with other domestic breeds as well as with wild boar.

## Figures and Tables

**Table 1:** Minimum Number of Individuals (MNI) of *S. scrofa* in each Stratigraphic Units (SU) of the two pits, and number of individuals sampled for aDNA extraction.

Pit Number	Chronology	SU	MNI	Sampled
1	ca.1400–1425 CE	1004	1	1
		1006	1	-
		1007	1	1
2	ca. 1450–1525 CE	2000	2	-
		2002	3	1
		2003	6	3
		2004	6	4
		2005	5	4
	ca. 1425–1450 CE	2006-2007-2008	5	5
		2009-2010-2011	3	2

**Table 2:** Number of individuals, pit number, stratigraphic units (SU), skeletal elements, age (based on [35]), DNA quantification with two different extraction protocol (ng/µL), and PCR results (DP: Domestic Pig; WBO: Wild BOar) are reported for the 21 samples identified in Table 1.

Pit Number	Sample ID	SU	Skeletal Element	Age	DE1 ^a^ (ng/µL)	DE2 ^b^ (ng/µL)	PCR1 ^c^	PCR2 ^d^	PCR3 ^e^
1	G8	1004	4th metatarsal	<24 months	0	6	DP(C/C)		
1	G9	1007	Mandible, right	31–35 months	6		DP(C/C)	DP(T/T)	F
2	G21	2008	Maxilla, left	7–11 months	0	2	DP(C/C)		
2	G24	2008	Mandible, right	2–4 months	0	3	CINTA(T/T)	DP(T/T)	
2	G161	2002	Maxilla, right	>4 years	3	7	CINTA(T/T)		
2	G182	2003	Cranial fragment, right	Undetectable	0	3	DP(C/C)	WBO(T/T)	
2	G192	2003	Mandible, left	31–35 months	3		DP(C/C)		
2	G206	2003	Mandible, left	0–4 months	4		DP(C/C)	DP(T/T)	
2	GB	2004	3rd metatarsal, left	>24 months	3		DP(C/C)		
2	G262	2004	Maxilla, right	7–11 months	4		DP(C/C)	DP(T/T)	M
2	G264	2004	Maxilla, left	7–11 months	4		DP(C/C)	WBO(C/C)	F
2	G286	2004	Maxilla, right	3–4 years	2		DP(C/C)	DP(T/T)	F
2	GC	2005	Femur, left	1–2 years	11		DP(C/C)	DP(T/T)	F
2	GD	2005	Mandible, left	0–4 months	4.5	7	CINTA(C/T)		
2	G133	2005	Mandible, left	3–4 years	3	4	CINTA (C/T)	WBO(C/C)	F
2	G198	2005	Mandible, left	0–4 months	2		DP(C/C)	DP(T/T)	F
2	G184	2006	Mandible, left	0–4 months	1		DP(C/C)	DP(T/T)	F
2	G23	2008	Mandible, right	0–4 months	4.5		DP(C/C)	WBO(C/C)	M
2	G27	2008	Mandible, left	>4 years	5.5		DP(C/C)	DP(T/T)	F
2	G4	2009	Mandible, left	7–11 months	4		DP(C/C)	DP(T/T)	F
2	G1	2011	Mandible, right	0–4 months	4.5		DP(C/C)	DP(T/T)	

^c^ PCR1: g.43597545C>T *KIT* SNP (CINTA vs. DP); ^d^ PCR2: SNP rs81460129 (WBO vs. DP); ^e^PCR3: sexing; ^a^ DE1: DNA extraction protocol 1 (Yang’s protocol, [46]); ^b^ DE2: DNA extraction protocol 2 (Dabney’s protocol [47,48]).
